# Phylogenetic conservatism and trait correlates of spring phenological responses to climate change in northeast China

**DOI:** 10.1002/ece3.3207

**Published:** 2017-07-22

**Authors:** Yanjun Du, Jingru Chen, Charles G. Willis, Zhiqiang Zhou, Tong Liu, Wujun Dai, Yuan Zhao, Keping Ma

**Affiliations:** ^1^ State Key Laboratory of Vegetation and Environmental Change Institute of Botany Chinese Academy of Sciences Beijing China; ^2^ The Key Laboratory of Forest Plant Ecology of Ministry of Education Northeast Forestry University Harbin China; ^3^ Harvard University Herbaria Cambridge MA USA; ^4^ School of Forestry Northeast Forestry University Harbin China; ^5^ South China Botanical Garden Chinese Academy of Sciences Guangzhou China

**Keywords:** climate change, flowering phenology, functional group, leaf‐out phenology, phylogenetic signal

## Abstract

Climate change has resulted in major changes in plant phenology across the globe that includes leaf‐out date and flowering time. The ability of species to respond to climate change, in part, depends on their response to climate as a phenological cue in general. Species that are not phenologically responsive may suffer in the face of continued climate change. Comparative studies of phenology have found phylogeny to be a reliable predictor of mean leaf‐out date and flowering time at both the local and global scales. This is less true for flowering time response (i.e., the correlation between phenological timing and climate factors), while no study to date has explored whether the response of leaf‐out date to climate factors exhibits phylogenetic signal. We used a 52‐year observational phenological dataset for 52 woody species from the Forest Botanical Garden of Heilongjiang Province, China, to test phylogenetic signal in leaf‐out date and flowering time, as well as, the response of these two phenological traits to both temperature and winter precipitation. Leaf‐out date and flowering time were significantly responsive to temperature for most species, advancing, on average, 3.11 and 2.87 day/°C, respectively. Both leaf‐out and flowering, and their responses to temperature exhibited significant phylogenetic signals. The response of leaf‐out date to precipitation exhibited no phylogenetic signal, while flowering time response to precipitation did. Native species tended to have a weaker flowering response to temperature than non‐native species. Earlier leaf‐out species tended to have a greater response to winter precipitation. This study is the first to assess phylogenetic signal of leaf‐out response to climate change, which suggests, that climate change has the potential to shape the plant communities, not only through flowering sensitivity, but also through leaf‐out sensitivity.

## 1 INTRODUCTION

The biological fingerprint of global warming has been recognized in organisms and communities around the world (Parmesan, 2006; Root et al., 2003). Spring phenological observations (leaf‐out and flowering timing) provide one of the most sensitive biological indicators of climate change (Peñuelas & Filella, 2001; Schwartz, 1999). The study of phenology has thus become an important tool for understanding and predicting the impacts of climate change on communities and diversity from local to global scales (Cleland, Chuine, Menzel, Mooney, & Schwartz, 2007; Inouye, 2008; Pau et al. 2011; Willis, Ruhfel, Primack, Miller‐Rushing, & Davis, 2008).

A growing number of studies have revealed an earlier onset of spring phenology in the northern hemisphere (CaraDonna, Iler, & Inouye, 2014; Everill, Primack, Ellwood, & Melaas, 2014; Morin et al., 2010; Parmesan, 2007; Polgar & Primack, 2011). One global meta‐analysis of both plants and animals found 62% of species displayed trends toward spring advancement across multiple phenophases (Parmesan & Yohe, 2003). Another meta‐analysis of more than 100,000 phenological time series of 542 European plants found 78% of the species exhibited a trend of advancing leaf‐out and flowering time (Menzel et al., 2006). More recently, a meta‐analysis restricted to China found that 91% of the spring/summer phenophases for both plants and animals examined exhibited an earlier trend (Ge, Wang, & Dai, 2015). Given these trends, being able to generalize how species phenologically respond to climate is necessary to be able to predict how climate change will impact plant communities around the world.

Phylogenies provide a potentially powerful tool for predicting generalizable patterns of species phenological response (Davis, Willis, Primack, & Miller‐Rushing, 2010). Flowering phenology is one of several plant functional traits that have been found to exhibit phylogenetic signal, that is, the tendency of closely related species to flower at the similar time during the year (Davies et al., 2013; Du et al., 2015; Willis et al., 2008; Wright & Calderon, 1995). There is little evidence that leaf‐out phenology also exhibits phylogenetic signal (Davies et al., 2013; Panchen et al., 2014). Whether phenological response to climate (i.e., climate‐driven phenological shifts) exhibits phylogenetic signal, however, remains an open question. This is due, in part, to the limited number of studies that have tested for phylogenetic signal in phenological response (Davis et al., 2010).

For instance, only a few studies have tested for phylogenetic signal in flowering time response to temperature (CaraDonna & Inouye, 2015; Davis et al., 2010; Mazer et al., 2013; Willis et al., 2008; Wolkovich et al., 2013). No study to date has tested for phylogenetic signal in leaf‐out response to temperature or precipitation. Furthermore, among the few studies reporting phylogenetic signal in flowering time response to temperature, results are inconsistent. Willis et al. (2008) and Davis et al. (2010) reported that flowering time response to temperature from two temperate plant communities in the USA and UK exhibited phylogenetic signal. In contrast, Wolkovich et al. (2013) and CaraDonna and Inouye (2015) did not find phylogenetic signal in flowering response to temperature across several North American plant communities. It remains unknown whether these regional results are applicable to other geographic regions or other plant communities, or other phenological traits such as leaf‐out sensitivity (Davis et al., 2010).

In addition to phylogeny, another potential predictor of phenological response is functional group. Leaf‐out and flowering time have been found to be associated with several important functional groups including: growth form (Du et al., 2015; Molau, Nordenhäll, & Eriksen, 2005; Panchen et al., 2014); pollination syndrome (Du et al., 2015; Proctor, Yeo, & Lack, 1996); fruit type (Bolmgren & Lönnberg, 2005; Du et al., 2015); deciduousness (Du et al., 2015; Panchen et al., 2014); and native/non‐native status (Willis et al., 2010; Wolkovich et al., 2013). In contrast, only a few studies have directly evaluated whether phenological response to climate differs among plant functional groups (Calinger, Queenborough, & Curtis, 2013; Fitter & Fitter, 2002; Miller‐Rushing & Primack, 2008; Willis et al., 2008; Wolkovich et al., 2013). Among these studies, invasive status was a regular predictor of flowering time response, with non‐native species being far more responsive to temperature than native species overall (Willis et al., 2010; Wolkovich et al., 2013). Pollination syndrome was also associated with flowering time response, with wind‐pollinated species being more responsive to temperature than insect‐pollinated species (Calinger et al., 2013); whether these associations extend to leaf‐out sensitivity remain to be studied.

The spring phenologies at higher latitude where there are heavy snowfall in winter are primarily a consequence of two environmental events, the warm spring temperature and the disappearance of snowpack (Inouye & Wielgolaski, 2003). Climate change at higher latitude includes warming in growth season temperature and receiving more precipitation as rain instead of snow (Johnson, 1998). In this study, we test the degree to which phylogeny and different functional groups predict first leaf‐out date (FLD), first‐flowering date (FFD), and the sensitivity of these two phenological events to temperature and winter precipitation. We take advantage of 52‐year observational phenological dataset that includes 52 woody species from the Forest Botanical Garden of Heilongjiang Province, China. Specifically, we investigate the following questions: *(1) Do FLD and FFD exhibit phylogenetic signal? (2) Are FLD and FFD sensitive to temperature and winter precipitation? (3) Does the sensitivity of FLD and FFD to temperature and precipitation exhibit phylogenetic signal? (4) Are FLD and FFD and their sensitivity to climate associated with different functional groups including*: native status, pollination syndrome, fruity type, and leaf‐out/flowering time?

## 2 METHODS

### 2.1 Study site

Phenology data were collected at the Forest Botanical Garden of Heilongjiang Province in the city of Harbin (Northeast China; 45.7°N,126.6°E). During 1951–2012, the annual mean temperature is 4.25°C, and extreme temperatures have ranged from −42.6°C to 39.2°C, and the mean yearly maximum and minimum temperatures are 23.0°C in July and −18.4°C in January, respectively. This area has an annual mean precipitation of 524 mm, and receives most of this precipitation (77%) during summer and early autumn (June–September). The winter precipitation refers to snowfall rather than rainfall from last November to March in our study site, and serves as a proxy for snowmelt out date. There was no irrigation in the garden, so winter precipitation could provide water plant needed to growth in early spring. The elevation ranges between 136 and 155 m.

### 2.2 Phenology data

The phenological data for this study were from the Chinese Phenological Observation Network, which was established in 1963. More than 170 woody species have been monitored for first leaf‐out date and first‐flowering date since 1963 in the Forest Botanical Garden. Botanical gardens are good sites for studying the effects of climate change on phenology (Primack & Miller‐Rushing, 2009; Zohner & Renner, 2014). Most of the trees were transplanted more than 30 years ago (personal communication with employed gardeners). There were 235 native species (41 families and 95 genera) and 97 introduced species documented in Heilongjiang province (Zhou, 1986). The FLD and FFD are defined as the date when individual plant of a given species unfolded the first young leaves and the first full flower, respectively. Unfortunately, data collection for the site has not been continuous. Observations were stopped from 1966 to 1972 and also throughout 1989–2002 due to the lack of financial support. Therefore, data for this study cover the periods 1963–1966, 1973–1989, and 2003–2014. In addition, there are some missing data points in a few years for certain species.

We exclude species with fewer than 10 years of data for flowering and leaf‐out in order to meet the minimum sample size for statistical analysis (Lessard‐Therrien, Davies, & Bolmgren, 2013). In total, 52 species, belonging to 20 families and 40 genera, had sufficient data for flowering phenology, and 50 species, belonging to 20 families and 39 genera, had sufficient data for leaf‐out phenology (Table S1). The number of annual observations per individual species varied from 10 to 27 (mean = 18; Table S1).

### 2.3 Climate data and climate sensitivities

Monthly climate data, including mean temperatures and precipitation, were obtained from the Harbin Meteorological station, located in the Forest Botanical Garden where we conducted the phenology monitoring. Over the course of our study period (1963–2014), the mean annual temperature has increased by 0.5°C per decade in this garden (Chen et al., 2017).

We used mean month temperatures from 1963 to 2014 and winter precipitation (sum of precipitation in November, December, January, February, and March) to represent snow cover in our analyzes that are proved to be important for spring phenology in temperate areas (Inouye, 2008). Similar climate indexes have been used in other high elevation phenology studies (CaraDonna & Inouye, 2015).

The relevant periods for leaf unfolding and flowering are typically one to three months prior to the phenological events (Beaubien & Freeland, 2000; Fu et al., 2015; Menzel et al., 2006) and can differ among species and locations. However, fall temperatures were also found to affect flowering times (e.g., Fitter, Fitter, Harris, & Williamson, 1995; Miller‐Rushing & Primack, 2008; Sparks & Carey, 1995). In previous studies, some have used single month temperature in the preceding 11 months (e.g., Miller‐Rushing & Primack, 2008), while others have used mean temperature of the preceding two months (e.g., Beaubien & Freeland, 2000), and still some utilize temperatures of successive 3‐month intervals beginning in August of preceding year (e.g., Cook, Wolkovich, & Parmesan, 2012; Mazer et al., 2013). Therefore, it is necessary and best to run a different combination of models to know which months are more suitable for most species in a study site.

The phenological response for each species to temperature was quantified as the slope of a linear regression model of leaf‐out/flowering date versus temperature. Linear regression to study the phenological response to temperature and precipitation has been widely used in previous studies (e.g., Abu‐Asab, Peterson, Shetler, & Orli, 2001; Calinger et al., 2013; Lesica & Kittelson, 2010; Menzel et al., 2006; Miller‐Rushing & Primack, 2008). We used single month temperature in the preceding months to leaf‐out/flowering beginning in August of preceding year, average temperature of the preceding two months or average temperature of successive 3‐month intervals beginning in August of preceding year. We selected the best model for each species based on Akaike information criterion (AIC).

The values for phenological response to winter precipitation were quantified as the slope of a linear regression model of leafing/flowering date versus winter precipitation.

Multiple linear regressions were conducted to examine whether phenology was influenced by the interaction of precipitation and temperature:

FLD/FFD=temperature+precipitation+temperature×precipitation

where temperature is the temperature combinations in the best model for most species, and precipitation is the winter precipitation.

As the phenological responses to temperature and precipitation were negative for most species, reference to a “more” or “greater” response refers a steeper negative slope, that is, a species with greater flower sensitivity flowered earlier in warmer years and less snowy years.

### 2.4 Functional groups

Each species was characterized based on pollination syndrome (wind vs. animal), origin (native vs. non‐native to the forest vegetation type of Heilongjiang [see below]), fruit type (fleshy vs. nonfleshy) (Table S1). Pollination syndrome and fruit type for each species were based on the description from *Flora of China* (http://frps.eflora.cn/), field observations, or judged from the morphology of the flowers. Showy flowers with conspicuous perianths were classified as animal‐pollinated species. Flowers with fewer or absent perianths, exposed stigmas with large surface area, high pollen quantity, and no nectar were classified into wind‐pollinated species. Fruit type was divided into fleshy fruits and nonfleshy fruits. Capsules, follicles, nutlets, samaras, nuts, dry arils, achenes, and cones were categorized as nonfleshy fruit. Berries, pomes, hesperidia, drupes, and sorosises were categorized as fleshy fruit. The main forest vegetation type of Heilongjiang province is Temperate Mixed Needleleaf and Deciduous Broadleaf Forest Region, which includes the main part of Heilongjiang province and Jilin province. Therefore, we defined a given species as “native” or “non‐native” if it was documented as “native” or “non‐native” in the local flora of the same vegetation region, including the *Flora of Heilongjiang* and *Flora of Jilin*. There were 43 native species and nine non‐native species in our study. There are many other phenological traits (e.g., leaf drop date, fruit date) which may be correlated with leaf‐out and flowering dates. However, data on leaf drop date and fruit date are unavailable and are not included them in this study. Naturally some functional types (e.g., pollination, fruit type) will not affect the growth of leaves because they are not organs for reproduction. Therefore, we only tested leaf‐out response to temperature/winter precipitation between non‐native and native species.

It is difficult to compare the phenological response of the best temperature model across species because they may not be responding to the same temperature cues during the same time of year. By making the response variable consistent, we can more easily compare species. Therefore, we calculated the phenological response to mean temperature of March, April, and May (MAM) for FLD and mean temperature of April and May (AM) for FFD as a standardized comparison of response across all species to spring temperatures. We chose these months because, for most species, FLD was correlated with March, April, or May temperatures, while FFD was correlated with April or May temperatures (see Fig. S4; Table S2). Thus, we calculated the phylogenetic signals and compared differences in the phenological response among functional groups to spring temperature (i.e., mean temperature of MAM for FLD and mean temperature of AM for FFD). This method of using a single temperature combination for each phenophase to study the difference among functional groups has been widely used to study phenological response to climate change (e.g., Beaubien & Freeland, 2000; Calinger et al., 2013; Miller‐Rushing & Primack, 2008).

To compare the difference of “phenological response to temperature/winter precipitation” between groups (non‐native vs. native, wind‐ vs. animal‐pollinated, dry‐ vs. fleshy‐fruited), we performed phylogenetic analyzes correcting for phylogenetic relationships to account for the potential effect of the shared evolutionary history of species. We used “pgls” in the package “*caper”* v0.5.2 (Orme, Freckleton, Thomas, Petzold, & Fritz, 2011) in R v2.15 (R Development Core Team 2014) to compare whether the phenological response were correlated with functional groups. For earliness of leaf‐out/flowering, “pgls” was used to investigate whether FFD and FLD were correlated with their corresponding phenological response to temperature and winter precipitation. A negative correlation would indicate that species with an early phenology are more sensitivity to interannual climate variation.

### 2.5 Phylogenetic tree

We built a phylogenetic tree based on DNA sequence data, which were collected from GenBank for 47 species (90% and 94% species for flowering and leafing phenology, respectively). We used the program *phyloGenerator2* (Pearse & Purvis, 2013) to download, align, and concatenate sequence data from GenBank for the following markers: *atp1*,* atpB*,* matR*,* rbcL*,* matK*,* psbA*,* ITS*,* ndhF*,* trnL‐trnF*. There were a total of 15,834 sites in the final concatenated matrix. Further alignment was performed by visual inspection. Using the concatenated matrix, partitioned for each marker, 100 maximum‐likelihood (ML) bootstrap phylogenies were constructed using RAxML‐HPC v8 on the CIPRES portal v3.3 (Miller, Pfeiffer, & Schwartz, 2010). A constraint tree was used to preserve known relationships. Time‐corrected branch lengths were than estimated for all 100 ml bootstrap trees based on seven major angiosperm node ages obtained from Bell et al. (Bell, Soltis, & Soltis, 2010) using the program *TreePL* (Smith & O'Meara, 2012).

We quantified the strength of phylogenetic signal in leaf‐out, flowering dates, and phenological response to spring temperature (MAM for FLD and AM for FFD) and winter precipitation using Blomberg's K (Blomberg, Garland, & Ives, 2003) using the “phylosignal” function in the package “picante” v0.2‐1(Kembel, Cowan, & Helmus, 2010) in R. Blomberg's K compares the observed distribution of tip data to expectations derived from a Brownian motion model of evolution, with expectation *K *=* *1.0 for a Brownian motion model and *K *=* *0 for absence of phylogenetic signal. We ran analyzes across all 100 bootstrap trees to account for phylogenetic variation.

To evaluate how one phenological event might help predict the timing of another, we performed phylogenetically regression analysis between all combinations of leaf‐out and flowering time response to temperature and winter precipitation using the “pgls” function in the package *caper* v0.5.2 in R (Orme, 2011).

## 3 RESULTS

Thirteen species leaf‐out in April and 37 species leaf‐out in May totaling 50 species under study (Fig. S1a). *Prinsepia sinensis* was the earliest to leaf‐out (average 16th April), following by *Sorbaria sorbifolia* and *Spiraea chamaedryfolia* (20th April). *Morus alba* was the latest to leaf‐out (19th May), followed by *Lespedeza bicolor* and *Xanthoceras sorbifolium* (17th May). The range of mean leaf‐out date was 33 days.

Species flowered from April to August, with most species flowering in May (33 species; Fig. S1b). Eight species flowered in June, seven in April, three in July, and only one in August. The earliest flowering species were *Ulmus pumila* on 17th April and *Ulmus davidiana* on 19th April. *Acanthopanax sessiliflorus* (Araliaceae) was the latest species, flowering on 1st August. The range of mean flowering time was 106 days.

### 3.1 Phylogenetic signal in leaf‐out and flowering phenology

Overall, leaf‐out date showed a marginally significant phylogenetic signal (*K *=* *0.460; *p *=* *.054), although still less than predicted by a Brownian motion model of trait evolution (expectation *K *=* *1). Further, there was a significant phylogenetic signal in flowering time (*K *=* *0.595; *p *=* *.002). These estimates were also robust to phylogenetic uncertainty based on analysis of 100 bootstrap DNA trees (Figs. S2 and S3).

### 3.2 Relationship between phenophases and climate variables

For leaf‐out date, 49 of 50 species (98%) were sensitive to temperature, exhibiting a significant negative relationship, thus leaf‐out dates being earlier in warmer years (Table S2). No species showed a significant positive relationship between leaf‐out date and temperature. The mean sensitivity of leaf‐out to temperature was −3.11 day/°C, while sensitivities ranged from −0.96 days/°C to −7.65 days/°C (Table S2). For 29 species, leaf‐out date was most sensitive to spring temperatures (Fig. S4a): Twelve species were most strongly associated with April temperatures; 10 species were most strongly associated with mean temperature of March and April; seven species were most strongly associated with the mean temperature of March, April, and May.

Forty‐eight of 52 species (92%) showed significant sensitivity in flowering time to temperature (Table S2). Flowering time, for most species, was correlated with spring temperatures (Table S2; Fig. S4b): Twenty‐one species were most strongly associated with April temperatures, while 13 species were most strongly associated with the mean temperature of April and May. Flowering time sensitivity to spring temperature did not necessarily depend on flowering time seasonality, with several late flowering species (June, July) exhibiting response to spring temperatures (Table S2). The average response of flowering time response to temperature was −2.87 day/°C (range: −6.42 days/°C to 5.54 days/°C). Only two species, *Acanthopanax sessiliflorus* and *Lonicera tatarinowii*, exhibited delayed flowering.

The correlations between FLD and FFD and winter precipitation were not strong (Table S2). The correlation coefficients of leaf‐out date of 43 species (86%) against winter precipitation were positive, but only four of them were significant (mean slope = 1.09). Correlation coefficients of leaf‐out date of the other seven species were negative, although none was significant (mean slope = −0.54). Similar to leaf‐out date, flowering time of 43 species (83%) correlated positively with winter precipitation, 12 significantly (mean slope = 1.43). The nine remaining species were negatively correlated with precipitation, but none was significant (mean slope = −0.72). In addition, the interaction of winter precipitation and temperature did not have significant influence on leaf‐out and flowering time for all but two species (Table S1).

The response of leaf‐out date to temperature predicted the response of flowering time to temperatures (β = 0.27, *R* ^2^ = .11, *p *= .015; based on the spring temperature model estimates of phenological response). Leaf‐out date response to winter precipitation did predict flowering time response to winter precipitation, such that species with more responsive FLD also had more responsive FFD (β = 0.62, *R* ^2^ **= .28, *p *<* *.001). Flowering time response to temperature and to winter precipitation was significantly correlated (β = .51, *R* ^2^ = .36, *p* < .001). However, no significant correlation was found between leaf‐out date response to temperature and winter precipitation (β = 0.13, *R* ^2^ = .01, *p *=* *.23).

### 3.3 Phylogenetic signal of phenological response

**Figure 1:**
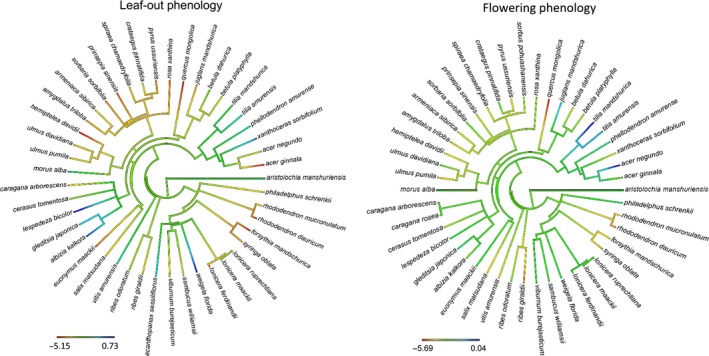
Phylogenetic distribution of phenological response to mean temperature of March, April, and May for leaf‐out date and mean temperature of April and May for flowering date on the ML tree topology. One outlier value in flowering response to temperature (−8.34 day/°C for *Acanthopanax sessiliflorus*) was not included in the plot in order to show the pattern clearly

The phenological response of leaf‐out date to spring temperature (MAM) exhibited marginally significant phylogenetic signal, and significant phylogenetic signal was found for flowering time response to spring temperature (AM; Table S2; Figure 1). Species in family Rosaceae (ten species in total) were particularly sensitive to spring temperature for both FLD and FFD, and all three species in family Ulmaceae and Saxifragaceae, and both species in family Ericaceae showed a strong response to temperature for both FLD and FFD (Table S2; Figure 1).

The response of leaf‐out date to winter precipitation exhibited no phylogenetic signal (Fig. S5), while flowering time response to winter precipitation exhibited phylogenetic signal (Table 1; Fig. S5). Consistent results were found when phylogenetic signal was tested across 100 bootstrap trees (Figs. S6–S9).

**Table 1:** Phylogenetic signal of phenological response to mean temperature of March, April, and May for leaf‐out date and to mean temperature of April and May for flowering date and to winter precipitation at the Forest Botanical Garden of Heilongjiang Province. *K* is the Bromberg's *K* value which measures the strength of phylogenetic signal, and *p* is the *p*‐value obtained by comparing the real data to a null distribution sampled from random permutations of the data

Phenology	Temperature		Winter precipitation	
	*K*	*p*	*K*	*p*
Leaf‐out response	0.502	.067	0.319	.371
Flowering time response	0.499	.019	0.588	.009

### 3.4 Functional groups

**Figure 2:**
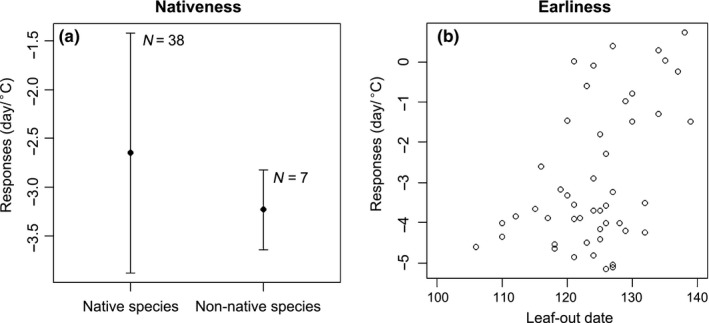
Results from phylogenetic generalized least squares (PGLS) comparing leaf‐out date response to mean temperature of March, April, and May across nativeness (native species vs. non‐native species) and mean leaf‐out date based on the PGLS results. The error bars stand for the standard errors. “*N*” is the sample size

For leaf‐out phenology, no significant differences of its response to MAM (Figure 2a; Table S3) and to winter precipitation (Fig. S10a; Table S3) were found between native species and non‐native species. The response of species to temperature for FLD was not significantly correlated with leaf‐out date (Figure 2b; Table S3). However, the response to winter precipitation for FLD was negatively correlated with leaf‐out date (Fig. S10b; Table S3), indicating that early season leaf‐out species have a greater response to winter precipitation than late season leaf‐out species.

**Figure 3:**
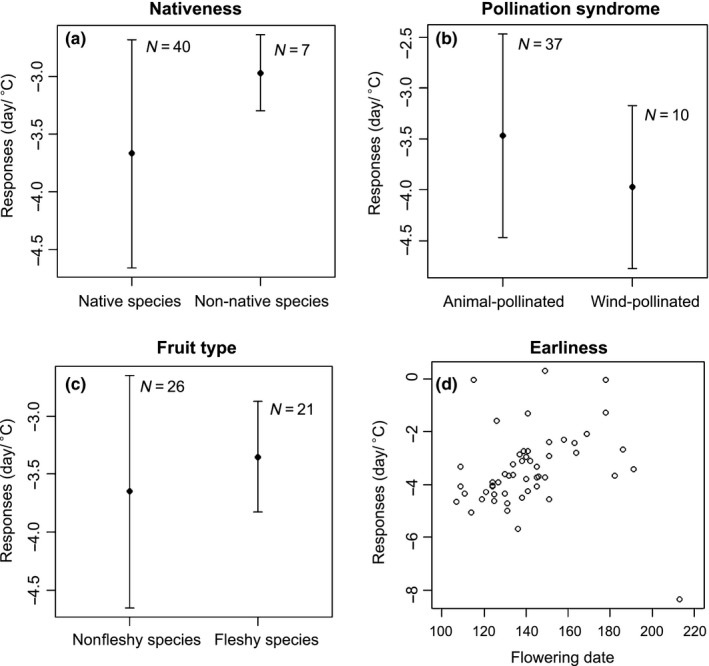
Results from phylogenetic generalized least squares (PGLS) comparing of flowering time response to mean temperature of April and May across multiple functional groups (nativeness, pollinator syndrome, and fruit type) and mean flowering time. The error bars stand for the standard errors. “*N*” is the sample size

For flowering phenology, there were no significant differences in species phenological response to AM or winter precipitation between wind‐pollinated species and animal‐pollinated species (Figure. 3b; Fig. S11b), between non‐fleshy‐fruited species and fleshy‐fruited species (Figure 3c; Fig. S11c), and between early flowering species and late flowering species (Figure 3d; Fig. S11d; Table S3). Native species tended to have a weaker flowering response to AM than non‐native species (Figure 3a; Table S3). However, no significant flowering response difference to winter precipitation was found between native species and non‐native species (Fig. S11a; Table S3).

## 4 DISCUSSION

This study is the first to assess whether the leaf‐out phenological response to climate factors exhibits phylogenetic signal and the first to document whether the leaf‐out response to climate is different among plant functional groups. We found phylogeny to be significant predictor of both phenological timing, as well as, phenological response to climate in this Botanical garden. In contrast, with few exceptions, we found most functional groups to be poor predictors of phenological response to climate.

### 4.1 Relationship between phenophases and climate variables

More than ninety percent of species exhibited a significant correlation between leaf‐out/flowering and seasonal temperature variation. Our study confirms that plant phenology is a sensitive indicator of temperature change (Cleland et al., 2007; Menzel et al., 2006). This is consistent with phenological studies in Asia (Chen, An, Inouye, & Schwartz, 2015; Dai, Wang, & Ge, 2014; Ge et al., 2015), Europe (Fu et al., 2014; Menzel et al., 2006; Vitasse et al., 2011), and North America (Abu‐Asab et al., 2001; Calinger et al., 2013; Miller‐Rushing & Primack, 2008; Willis et al., 2008; Wolkovich et al., 2013).

The average advance of leaf‐out and flowering to temperature is −3.11 and −2.87 day/°C, respectively, with large interspecific variation. Our observed average flowering response to temperature was weaker than a similar study in Xi'an, China (5.99 day/°C; range: −2.84 to −11.44; Dai, Wang, & Ge, 2013a), which is 11° latitude south of our study site. This does not support the expectation that the phenological response should be stronger as the result of a stronger seasonal variation at higher latitude in the Northern hemisphere (Jones et al., 2012). The tendency for spring phenophases of woody plants to be stronger at lower latitudes than at higher latitudes has been observed in other studies in China (Chen & Xu, 2012; Dai, Wang, & Ge, 2013b; Dai et al., 2014; Ge et al., 2015), and suggests that, in certain cases, latitude may not be a strong predictor of species phenological response to temperature. Beyond the average community response, the large interspecific variation in response will also likely affect the structure composition of plant communities as the climate warms (Cleland et al., 2007). Species in our study less able to respond phenologically to climate change could significantly decline in abundance, putting them at great risk of extirpation and even extinction (Davis et al., 2010; Willis et al., 2008).

Most species in this study leaf‐out and flower in May and are sensitive to temperature in the previous month or a combination of previous months. This is consistent with other studies that have found temperatures of the month preceding the phenological event to be the best predictors of phenology, rather than the temperatures of the month in which the event occurred (Fitter & Fitter, 2002; Miller‐Rushing & Primack, 2008).

We did not find that winter precipitation had a significant impact on spring phenology for most species tested. This is consistent with previous studies (e.g., Abu‐Asab et al., 2001; Sparks, Huber, & Croxton, 2006) that reported no significant correlation between flowering time and precipitation. However, our results are contrary to Lesica and Kittelson (2010) work on herbaceous broad‐leaved species conducted in Montana, USA (46°N), which showed that mean first‐flowering date advanced with a decline in winter precipitation (mainly in the form of snow). One reason is higher precipitation in winter at this site meant greater snowpack, later snowmelt and hence later flowering. This association has been reported for mountain wildflowers; snowmelt date was the primary determinant of flowering time in *Delphinium barbeyi* and *Androsace septentrionali*s (Inouye, Morales, & Dodge, 2002; Inouye, Saavedra, & Lee‐Yang, 2003). Alternatively, increased soil moisture due to greater snowpack could be a mechanism driving earlier phenology (Wolkovich et al., 2013). Therefore, whether the winter snowpack advances or delays spring phenology depends on the relative importance of its advantage and disadvantage.

Interestingly, we found the response of leaf‐out date to spring temperature could predict the response of flowering time to spring temperatures. In addition, the response of leaf‐out date to winter precipitation could predict the response of flowering time to winter precipitation. Furthermore, the flowering phenological response to temperature could predict the flowering response to winter precipitation. This finding suggests that mechanisms of sensing climate for spring phenology may not be independent, with species having stronger responses to abiotic factors for leaf‐out phenology under the climate change scenario tend to have stronger responses for flowering phenology, indicating that the response to climate of one phenological event might help predict that of another.

### 4.2 Phylogenetic signal in leaf‐out and flowering phenology

Consistent with other studies, we found evidence in support of phylogenetic signal in leaf‐out date and first‐flowering date (Bolmgren & Cowan, 2008; Du et al., 2015; Panchen et al., 2014; Wright & Calderon, 1995), revealing that more closely related taxa tend to commence spring phenology at similar times during the growing season. The phylogenetic signal in spring phenology may be due in part to its correlation with other phylogenetically conserved traits (Panchen et al., 2014). Closely related species tend to share similar physiological and morphological traits, such as flower size, shape, scent, and nectar production which can determine the attraction and success of pollination, and, thus might influence when species flower (Westoby, Leishman, & Lord, 1995). In addition, genetically based trait conservatism may play a role–plant physiology might dictate species response to a particular environmental cue, and hence, closely related species would be expected to share similar physiologies and sensitivities (Harvey & Pagel, 1991).

We also find the evidence for phylogenetic conservatism in the response to temperature for both leaf‐out and flowering phenology. These results are suggestive of a mediating phenological response to climate for certain traits among closely related species and a conserved physiological response to those abiotic conditions (Davies et al., 2013; Davis et al., 2010). Cautions should be taken, however, when inferring such broad evolutionary trends from community level patterns. Indeed, prior studies have found contrasting evidence for phylogenetic signal in flowering time response to temperature (CaraDonna & Inouye, 2015; Davis et al., 2010). Our study provides a similar contrast with regard to flowering time response to temperature and precipitation, with the added novelty–and complexity–of a differing patterns of phylogenetic signal in leaf‐out response. In short, while such phylogenetic patterns are relevant to the given community in question, there does not appear to be a broader phylogenetic pattern of phenological response to climate. Of course, the small numbers of studies that have looked at phenological response to climate limit our ability to make such broad inferences, as well as, identify the underlying mechanisms that are responsible for the differences in phylogenetic signal across communities.

The clades that tended to drive the patterns of phylogenetic signal that we observed in phenological responses to spring temperature included the Rosaceae, Ulmaceae, Saxifragaceae, and Ericaceae families. All species from these families were relatively responsive to temperature for leaf‐out and flowering time. While this is relatively small sample of species to infer broad scale patterns of the evolutionary history phenology in these families, it is notable that all four of these families have been shown to have conserved phenological behavior in other studies in the northern hemisphere (e.g., Panchen et al., 2014; Willis et al., 2008).

The difference in between the lack of phylogenetic signal in the FFD response to winter precipitation and the presence of phylogenetic signal in FLD may be due to the lack of any real correlation between FLD and winter precipitation itself, with only four species having a significant correlation. As this is the first study to explore the phylogenetic signal in leaf‐out response to winter precipitation, it remains to be tested whether this is a general pattern in other regions and for other species.

Although we only studied 52 woody species and it is a small sample size, there were 235 native species documented in this province (Zhou, 1986) and the sample of species in our study represent a reasonable proxy for the local flora. However, many families have only one or two species in our study, therefore, extrapolation beyond the species we looked to draw any phylogenetic conclusions would be unwise.

### 4.3 Functional groups

It has been found that temperate species that flower early in the growing season (e.g., spring ephemerals) are more responsive to temperature (Calinger et al., 2013; Fitter & Fitter, 2002; Menzel et al., 2006) and is likely an adaptation to the higher temperature variability in spring months (Menzel et al., 2006). However, we found that neither early season leaf‐out nor flowering species did not show greater phenological response to temperature than late season species. This may reflect one possible strategy that early season species at higher latitudes are unable to advance leaf‐out and flowering dates because abiotic conditions constrain them (e.g., snowfall continues well into April at our site). For example, one study showed that flower buds of a few species are sensitive to frost in the Colorado Rocky Mountains, USA, and the earlier beginning of the growing season under global warming has exposed them to more frequent mid‐June frost kills (Inouye, 2008). We also found early season leaf‐out species tend to have a greater phenological response to winter precipitation, showing that snowfall in winter would benefit early leaf‐out species by increased soil moisture due to greater snowpack.

In recent years, a few studies have found non‐native species tend to be more responsive to temperature than native species (e.g., Hulme, 2011; Willis et al., 2010; Wolkovich et al., 2013). One of the dominant explanations is the “vacant phenological niche model,” which suggests that the most successful invaders are phenologically more flexible and can occupy open temporal niche space near the start or the end of the growing season (Wolkovich et al., 2013). Our study is consistent with these findings for FFD. Furthermore, this result is congruent with the conceptual framework that opportunistic taxa (pioneers or exotic species) adopt a more risky strategy and may profit more from a warmer climate than native late‐successional species which show a more “conservative” and more complex response, with a large chilling requirement and photoperiod response (Körner & Basler, 2010).

In contrast, we found no significant difference between non‐native species and native species for leaf‐out response to temperature, and for both FLD and FFD response to winter precipitation. The lack of a difference between non‐native and native species could arise from the compressed growing season in our higher latitude site, where most species leaf‐out and flower within a relatively short window, leaving little open temporal niche space to occupy. In support of this hypothesis, we did not find significant differences between non‐native and native species for leaf‐out date and flowering time. Wolkovich et al. (2013) also reported no difference in phenological response to temperature between non‐native and native species for two grassland communities (in contrast, they did find a significant difference in three mesic temperate communities). Instead, Wolkovich et al. (2013) found non‐native and native species diverged in their response to precipitation, a pattern we did not find. This suggests that phenological response to climate may not be a globally advantageous trait for successfully non‐native species, and that, specifically, phenological responsiveness to temperature may be limited to mesic temperate communities (Davis et al., 2010; Willis et al., 2010; Wolkovich et al., 2013).

The association between phenological response and pollination syndrome remains largely untested and the few studies that have investigated it show mixed results. Calinger et al. (2013) found spring flowering wind‐pollinated species to be more phenologically responsive than animal‐pollinated species in north‐central North America. In contrast, Dai et al. (2013a) found greater advancement of flowering with increased temperature among biotically pollinated species with a weaker response among wind‐pollinated species. We found no significant difference between wind‐pollinated species and animal‐pollinated species in phenological changes in response to both temperature and winter precipitation, indicating that at higher latitudes, wind‐pollinated species, and animal‐pollinated species could convergence on their response to climate change.

Although few studies were carried out to compare the phenology between non‐fleshy‐fruited species and fleshy‐fruited species (e.g., Bolmgren & Lönnberg, 2005; Du et al., 2015), our research is the first study to assess the phenological response among fruit types, and found that non‐fleshy‐fruited species showed similar phenological response to both temperature and winter precipitation as did fleshy‐fruited species. The mechanisms behind this pattern need further investigation.

## 5 CONCLUSIONS

Our study confirms that both spring phenology (both leaf‐out and flowering phenology) and their responses to spring temperature are constrained by phylogeny, suggesting that phylogenetic relatedness is one potential tool that could be used to predict species response to future climate change in this region. Furthermore, the phylogenetic signal in phenological sensitivity means that future climate change may contribute to an increased loss of phylogenetic diversity in this region, similar to what has been observed in other ecosystems (Willis et al., 2008). While we do not have direct evidence of such a link here, we demonstrate one important component of this process. Future work will be needed to investigate the relationship between sensitivity and local species decline.

## CONFLICT OF INTEREST

None declared.

## AUTHOR CONTRIBUTIONS

YJD, JRC, ZQZ and KPM developed the original idea; YJD, JRC and TL conducted the fieldwork. YJD, CGW, JRC, YZ, and WJD analyzed the data. YJD and CGW wrote the first draft and all authors contributed to revisions.
