# Supplementary material for: Phylogenetic conservatism and trait correlates of spring phenological responses to climate change in northeast China
Source: Ecol Evol. 2017 Jul 22;7(17):6747–57. doi: 10.1002/ece3.3207 (PMC5587463; doi:10.1002/ece3.3207)
Supplement: Supplementary file 1 [file ECE3-7-6747-s001.doc]

**Figure S1.**  Frequency of number of species per month for (a) first Leaf-out date and (b) first flowering date. Numbers above the bar are sample size.

**Figure S2.** The frequency of (a) Blomberg’s K and (b) P values for leaf-out times by running across all 100 bootstrap trees to account for phylogenetic variation. The red lines are values using the best maximum-likelihood tree.

**Figure S3.** The frequency of (a) Blomberg’s K and (b) P values for flowering dates by running across all 100 bootstrap trees to account for phylogenetic variation. The red lines are values using the best maximum-likelihood tree.

**Figure S4.** Frequency of the number of species with significant responses to temperature for (**a**) leaf-out date and (**b**) first flowering date. “1”-January; “1+2+3”-average temperature of January, February and March; “2+3”- average temperature of February and March. Numbers above the bar are sample size.

Figure S5. Phylogenetic distribution of phenological response to winter precipitation for leaf-out date and first flowering date on the ML tree topology.

**
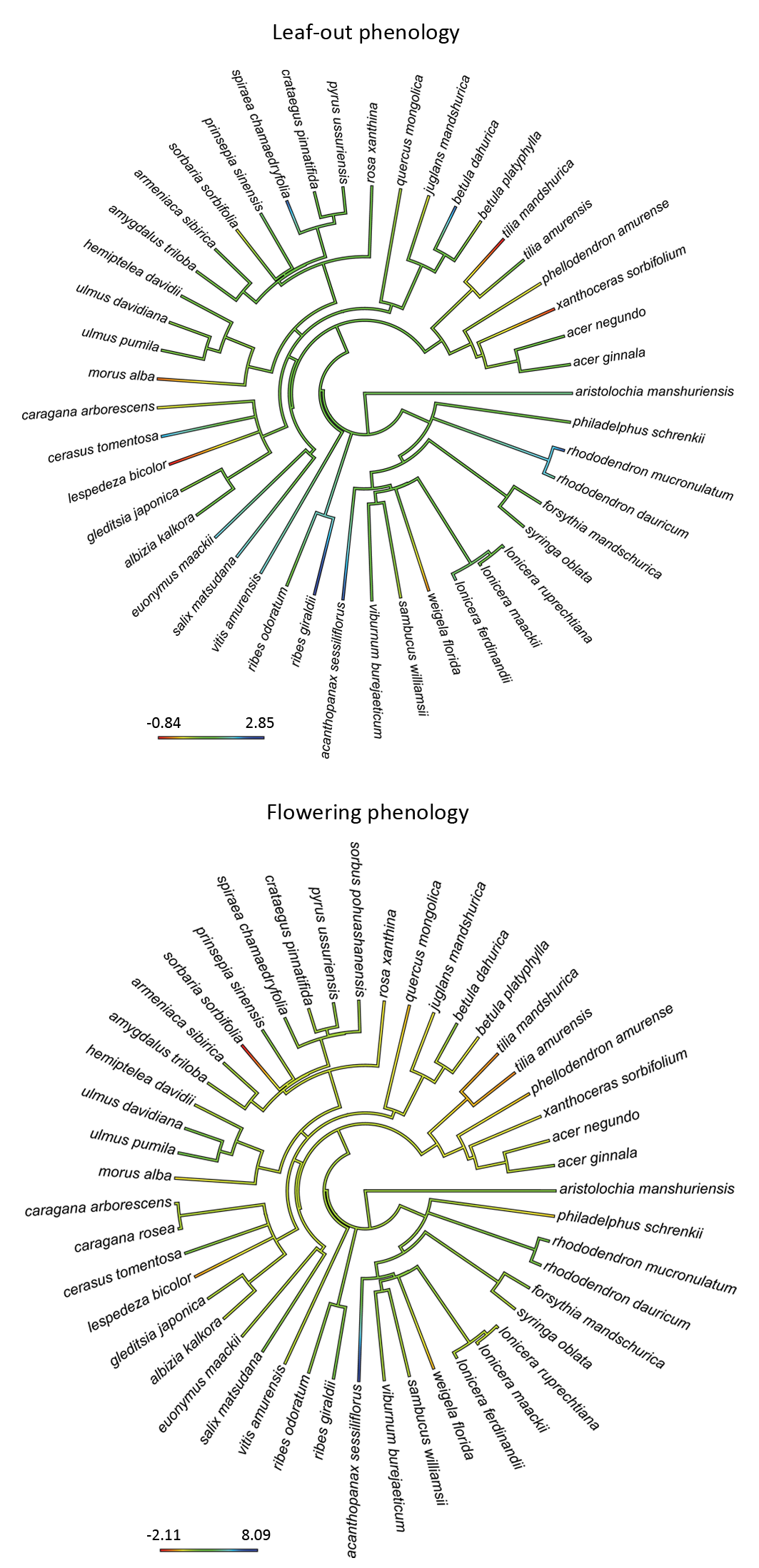
**

Figure S6. The frequency of (a) Blomberg’s K and (b) P values for phenological response of leaf-out times to mean temperature of March, April, and May by running across all 100 bootstrap trees to account for phylogenetic variation. The red lines are values using the best maximum-likelihood tree.


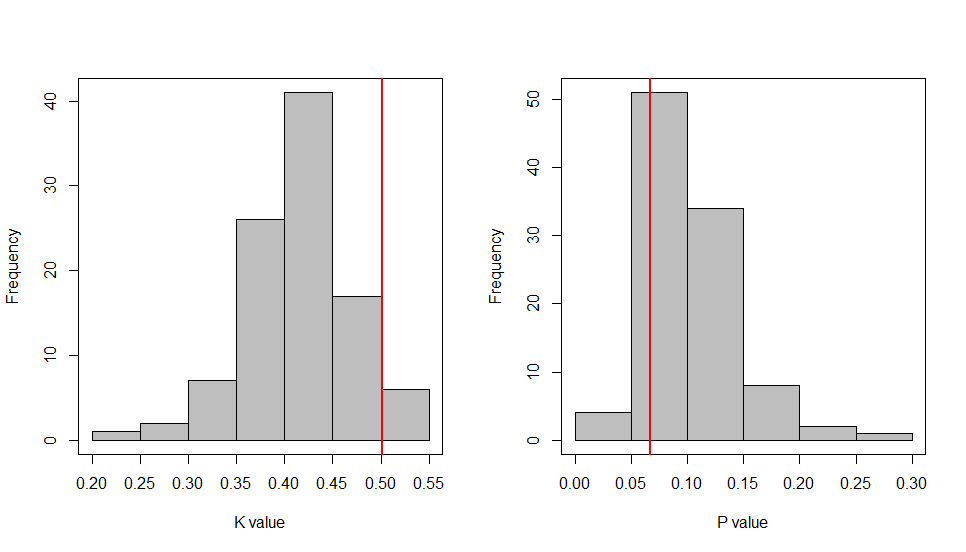


Figure S7. The frequency of (a) Blomberg’s K and (b) P values for phenological response of flowering dates to mean temperature of April and May by running across all 100 bootstrap trees to account for phylogenetic variation. The red lines are values using the best maximum-likelihood tree.


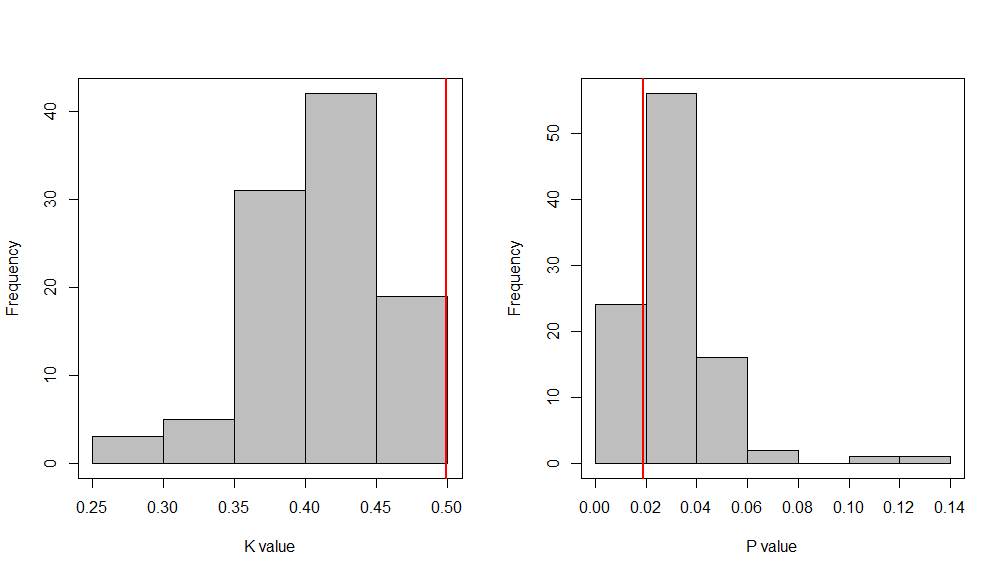


Figure S8. The frequency of (a) Blomberg’s K and (b) P values for phenological response of leaf-out times to winter precipitation by running across all 100 bootstrap trees to account for phylogenetic variation. The red lines are values using the best maximum-likelihood tree.

Figure S9. The frequency of (a) Blomberg’s K and (b) P values for phenological response of flowering time to winter precipitation by running across all 100 bootstrap trees to account for phylogenetic variation. The red lines are values using the best maximum-likelihood tree.

Figure S10. Results from phylogenetic generalized least squares (PGLS) comparing leaf-out date response to winter precipitation across multiple functional groups (nativeness, pollinator syndrome, and fruit type) and mean leaf-out date based on the PGLS results. The error bars stand for the standard errors. ‘N’ is the sample size.


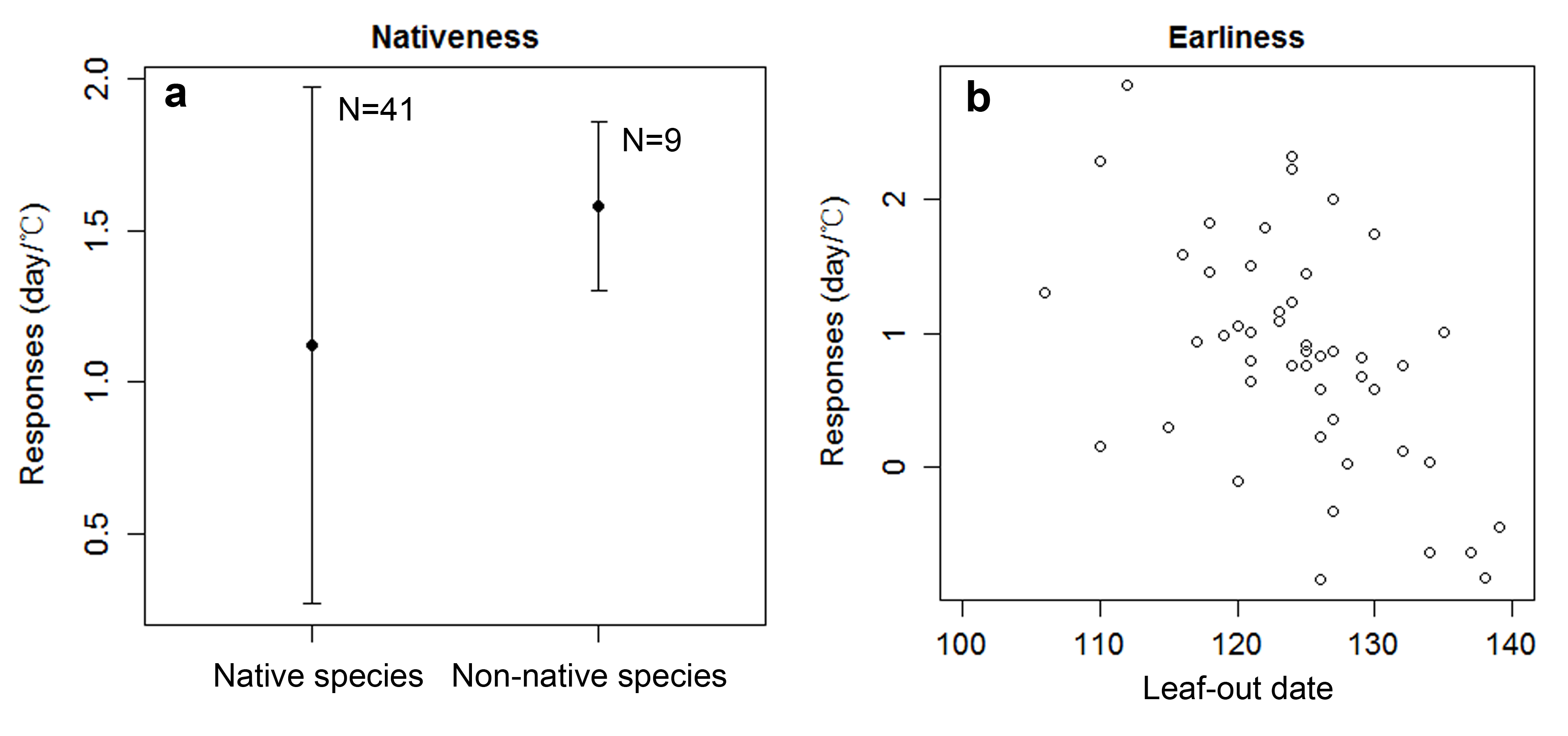


Figure S11. Results from phylogenetic generalized least squares (PGLS) comparing of flowering time response to winter precipitation across multiple functional groups (nativeness, pollinator syndrome, and fruit type) and mean flowering time. The error bars stand for the standard errors. ‘N’ is the sample size.


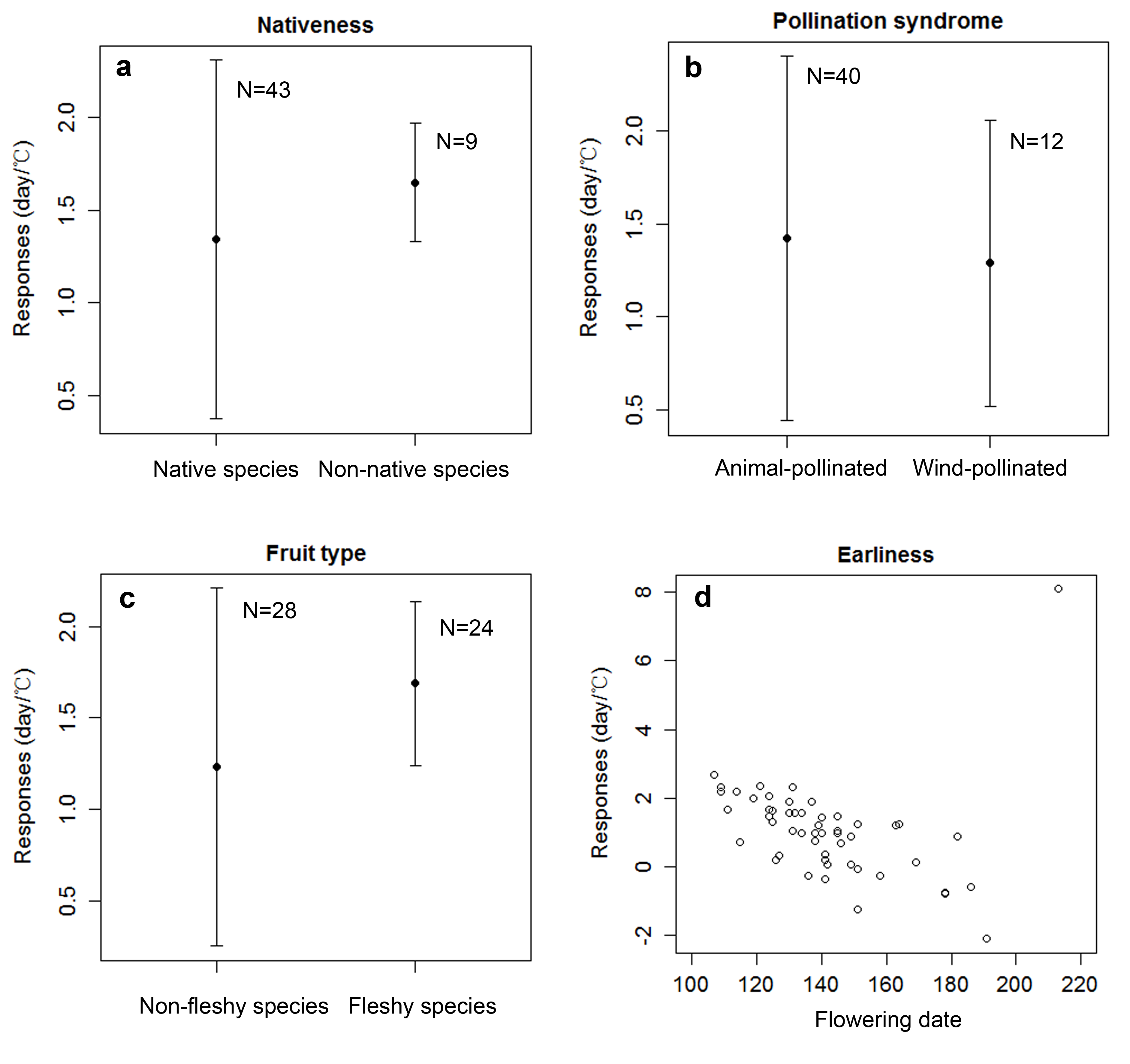


Table S1. The basic information for each species and the parameter estimates for the regression models. ‘N’ is the number of individuals monitored phenology. ‘AIC.leaf’ and ‘AIC.flower’ are AIC values for each simple regressions between leaf-out/flowering date and the temperature (the best temperature model); ‘Interaction.L’ and ‘Interaction.F’ are the coefficients of the models testing whether the interaction between temperature and winter precipitation influenced the leaf-out/flowering date; ‘AIC.interaction.L’ and ‘AIC.interaction.F’ are AIC values for the multiple linear regressions that are conducted to examine whether phenology was influenced with both precipitation and temperature. ** 0.001<P<0.01, * 0.01 < P < 0.05; . 0.05 < P < 0.10

| Species | Fruit type | Pollination type | Nativeness | N | AIC.leaf | AIC.flower | Interaction.L | AIC.interaction.L | Interaction.F | AIC.interaction.F |  |
| --- | --- | --- | --- | --- | --- | --- | --- | --- | --- | --- | --- |
| *Acer ginnala* | Dry fruit | Animal | native | 19 | 107 | 110 | 0.43. | 107 | 0.04 | 113 |  |
| *Acer negundo* | Dry fruit | Animal | non-native | 15 | 86 | 77 | 1.42* | 85 | 0.44 | 80 |  |
| *Acanthopanax sessiliflorus* | Fleshy fruit | Animal | native | 13 | 76 | 101 | 0.21 | 77 | 1.49 | 94 |  |
| *Aristolochia manshuriensis* | Dry fruit | Animal | native | 12 | 79 | 74 | -0.27 | 83 | 0.57 | 73 |  |
| *Betula platyphylla* | Dry fruit | Wind | native | 17 | 104 | 92 | 0.63 | 106 | 0.35 | 92 |  |
| *Betula dahurica* | Dry fruit | Wind | native | 12 | 76 | 76 | 0.90** | 66 | 0.98** | 63 |  |
| *Lonicera tatarinowii* | Fleshy fruit | Animal | non-native | 18 | 107 | 98 | 0.28 | 108 | -0.13 | 101 |  |
| *Viburnum sargenti* | Fleshy fruit | Animal | native | 22 | 150 | 183 | 0.17 | 151 | 0.59 | 183 |  |
| *Sambucus williamsii* | Fleshy fruit | Animal | native | 20 | 143 | 146 | 0.67 | 144 | -0.07 | 149 |  |
| *Lonicera maackii* | Fleshy fruit | Animal | native | 25 | 130 | 126 | 0.18 | 131 | 0.15 | 127 |  |
| *Weigela florida* | Dry fruit | Animal | native | 20 | 129 | 103 | -0.63 | 132 | 0.05 | 104 |  |
| *Viburnum burejaeticum* | Fleshy fruit | Animal | native | 22 | 135 | 110 | 0.22 | 138 | 0.08 | 112 |  |
| *Lonicera ferdinandii* | Fleshy fruit | Animal | non-native | 13 | 80 | 63 | 0.49 | 81 | 0.28 | 61 |  |
| *Lonicera ruprechtiana* | Fleshy fruit | Animal | native | 13 | 78 | 73 | -0.11 | 81 | -0.73 | 73 |  |
| *Euonymus maackii* | Dry fruit | Animal | native | 19 | 108 | 137 | 0.25 | 111 | 1.5 | 135 |  |
| *Rhododendron dauricum* | Dry fruit | Animal | native | 17 | 100 | 90 | 0.48 | 102 | 0.35 | 91 |  |
| *Rhododendron mucronulatum* | Dry fruit | Animal | non-native | 12 | 66 | 63 | 0.44. | 65 | 0.31 | 65 |  |
| *Quercus mongolica* | Dry fruit | Wind | native | 16 | 167 | 110 | 0.21 | 167 | 0.88 | 110 |  |
| *Juglans mandshurica* | Fleshy fruit | Wind | native | 24 | 150 | 159 | 0.48 | 150 | -0.21 | 162 |  |
| *Lespedeza bicolor* | Dry fruit | Animal | native | 14 | 122 | 89 | 0.79 | 123 | 2.35 | 90 |  |
| *Albizia kalkora* | Dry fruit | Animal | native | 14 | 150 | 92 | 0.28 | 152 | 1.85 | 92 |  |
| *Gleditsia japonica* | Dry fruit | Animal | non-native | 12 | 79 | 84 | -0.08 | 83 | 1.63 | 87 |  |
| *Caragana arborescens* | Dry fruit | Animal | native | 24 | 135 | 133 | 0.14 | 136 | -0.06 | 135 |  |
| *Caragana rosea* | Dry fruit | Animal | native | 10 | NA | 52 | NA | NA | 0.07 | 56 |  |
| *Morus alba* | Fleshy fruit | Wind | native | 20 | 139 | 118 | -1.98. | 140 | -0.16 | 122 |  |
| *Syringa oblatavar.alba* | Dry fruit | Animal | native | 22 | 142 | 123 | -0.15 | 141 | 0.27 | 124 |  |
| *Forsythia mandschurica* | Dry fruit | Animal | non-native | 20 | 122 | 105 | 0.31 | 126 | -0.15 | 107 |  |
| *Ligustrum longipedicellatum* | Fleshy fruit | Wind | non-native | 14 | 101 | 76 | 0.18 | 105 | 0.84 | 76 |  |
| *Fraxinus mandschurica* | Dry fruit | Wind | native | 20 | 171 | 133 | -1.95 | 172 | 0.51 | 136 |  |
| *Syringa oblata* | Dry fruit | Animal | native | 27 | 145 | 154 | 0.35 | 148 | 0.33 | 156 |  |
| *Prinsepia sinensis* | Fleshy fruit | Animal | native | 17 | 112 | 110 | -0.06 | 112 | 0.32 | 89 |  |
| *Rosa xanthina* | Fleshy fruit | Animal | native | 10 | 50 | 59 | -0.39. | 44 | -0.14 | 54 |  |
| *Pyrus ussuriensis* | Fleshy fruit | Animal | native | 21 | 122 | 112 | 0.08 | 124 | 0.41* | 109 |  |
| *Armeniaca sibirica* | Fleshy fruit | Animal | native | 24 | 144 | 125 | 0.37 | 145 | 0.28 | 125 | |
| *Crataegus pinnatifida* | Fleshy fruit | Animal | native | 19 | 104 | 91 | 0.05 | 107 | 0.18 | 88 | |
| *Spiraea chamaedryfolia* | Dry fruit | Animal | native | 11 | 63 | 60 | 0.03 | 67 | 0.47. | 59 | |
| *Cerasus tomentosa* | Fleshy fruit | Animal | native | 12 | 63 | 66 | 0.21 | 66 | 0.06 | 69 | |
| *Amygdalus triloba* | Fleshy fruit | Animal | native | 25 | 144 | 126 | 0.26 | 148 | 0.25 | 127 | |
| *Sorbaria sorbifolia* | Dry fruit | Animal | native | 23 | 173 | 169 | -0.25 | 174 | -2.24 | 170 | |
| *Sorbus pohuashanensis* | Fleshy fruit | Animal | native | 11 | NA | 60 | NA | NA | 1.73. | 59 | |
| *Phellodendron amurense* | Fleshy fruit | Wind | native | 16 | 139 | 92 | -0.01 | 141 | -0.11 | 95 | |
| *Salix matsudana* | Dry fruit | Wind | native | 17 | 93 | 95 | 0.22 | 96 | 0.22 | 98 | |
| *Xanthoceras sorbifolium* | Dry fruit | Animal | native | 20 | 126 | 94 | -1.67 | 128 | 0.06 | 98 | |
| *Philadelphus schrenkii* | Dry fruit | Animal | native | 23 | 129 | 144 | 0.27 | 231 | 0.11 | 148 | |
| *Ribes giraldii* | Fleshy fruit | Animal | non-native | 12 | 69 | 78 | 0.27 | 71 | 0.71 | 79 | |
| *Ribes odoratum* | Fleshy fruit | Animal | non-native | 17 | 90 | 89 | 0.07 | 90 | 0.33 | 90 | |
| *Tilia mandshurica* | Fleshy fruit | Animal | native | 11 | 72 | 52 | -2.02 | 74 | 0.68 | 53 | |
| *Tilia amurensis* | Dry fruit | Animal | native | 15 | 115 | 79 | -0.06 | 119 | 0.13 | 83 | |
| *Hemiptelea davidii* | Dry fruit | Wind | native | 24 | 148 | 144 | 0.66 | 145 | 0.27 | 143 | |
| *Ulmus davidiana* | Dry fruit | Wind | native | 16 | 103 | 111 | 0.18 | 106 | -0.19 | 110 | |
| *Ulmus pumila* | Dry fruit | Wind | native | 26 | 131 | 168 | 0.31. | 129 | -0.24 | 166 | |
| *Vitis amurensis* | Fleshy fruit | Animal | native | 12 | 77 | 89 | 0.04 | 80 | 1.54 | 89 | |

**Table S2.** Leaf-out and flowering dates and response to climate variables for 52 species at the Forest Botanical Garden of Heilongjiang Province, Northeast China. ‘N’ is number of individuals monitored; ‘Date’ is Julian day of leaf-out or first flowering date; ‘Month’ is the month plant leaf-out or flowering; ‘Best.Temp’, ‘Mean.Temp’, and ‘Winter.Precip’ are phenological response to temperature (the slope values; day/°C; negative means phenology advances) in best regression model, phenological response to mean temperature of March, April, and May for leaf-out date and mean temperature of April and May for flowering date, and response to winter precipitation, respectively. *** indicates P<0.001, ** 0.001<P<0.01, * 0.01 < P < 0.05; . 0.05 <*P*< 0.10

|  |  | Leaf phenology | | | | | |  | Flowering phenology | | | | | |
| --- | --- | --- | --- | --- | --- | --- | --- | --- | --- | --- | --- | --- | --- | --- |
|  | | | | | |  | | | | | |
| Species | Family | Date | Month | Month of  response | Best.Temp | Mean.Temp | Winter.Precip |  | Date | Month | Month of  response | Best.Temp | Mean.Temp | Winter.Precip |
| *Acer ginnala* | Aceraceae | 127 | 5 | 4+3+2 | -3.93*** | -5.06*** | 0.87 |  | 145 | 5 | 4 | -2.69*** | -3.71*** | 1.48 |
| *Acer negundo* | Aceraceae | 120 | 4 | 4+3 | -2.64*** | -3.31*** | 1.06 |  | 115 | 4 | 4 | -3.05*** | -0.04 | 0.71 |
| *Acanthopanaxsessiliflorus* | Araliaceae | 124 | 5 | 12 | -1.89** | -0.66 | 2.38* |  | 213 | 8 | 1 | 5.54* | -8.34 | 8.09* |
| *Aristolochia manshuriensis* | Aristolochiaceae | 125 | 5 | 3 | -1.8** | -1.81 | 1.45 |  | 137 | 5 | 4+3 | -1.64* | -2.86 | 1.88* |
| *Betula platyphylla* | Betulaceae | 126 | 5 | 4+3 | -2.75*** | -3.58*** | 0.23 |  | 127 | 5 | 4+3+2 | -2.71*** | -3.91** | 0.33 |
| *Betula dahurica* | Betulaceae | 124 | 5 | 5+4 | -2.83** | -2.89** | 2.23. |  | 124 | 5 | 4 | -2.9** | -4.08 | 1.65 |
| *Lonicera tatarinowii* | Caprifoliaceae | 120 | 4 | 2+3 | -1.07* | -1.47* | -0.1 |  | 149 | 5 | 10+9 | 2.82** | 0.31 | 0.87 |
| *Viburnum sargenti* | Caprifoliaceae | 121 | 5 | 1 | -1.07* | 0.01 | 1.01 |  | 151 | 6 | 4 | -3.02. | -4.55 | -1.26 |
| *Sambucus williamsii* | Caprifoliaceae | 115 | 4 | 4+3+2 | -2.68** | -3.66*** | 0.3 |  | 134 | 5 | 4+3+2 | -1.94* | -3.22 | 0.97 |
| *Lonicera maackii* | Caprifoliaceae | 121 | 5 | 4 | -2.69*** | -3.55*** | 0.64 |  | 146 | 5 | 5+4 | -3.68*** | -3.68*** | 0.69 |
| *Weigela florida* | Caprifoliaceae | 127 | 5 | 2+1+12 | -1.77** | 0.4 | -0.33 |  | 141 | 5 | 5+4 | -2.72*** | -2.72*** | -0.38 |
| *Viburnum burejaeticum* | Caprifoliaceae | 117 | 4 | 4 | -2.69*** | -3.88*** | 0.94 |  | 139 | 5 | 4 | -1.83*** | -2.73*** | 1.21* |
| *Loniceraferdinandii* | Caprifoliaceae | 118 | 4 | 4 | -3.25*** | -4.64*** | 1.46 |  | 151 | 6 | 5+4 | -2.91** | -2.91*** | 1.23. |
| *Lonicera ruprechtiana* | Caprifoliaceae | 116 | 4 | 4 | -2.3** | -2.60* | 1.59. |  | 140 | 5 | 5+4 | -2.95** | -2.95** | 0.97 |
| *Euonymus maackii* | Celastraceae | 118 | 4 | 4+3 | -3.3*** | -4.55*** | 1.83 |  | 164 | 6 | 5+4 | -2.79 | -2.79 | 1.24 |
| *Rhododendron dauricum* | Ericaceae | 121 | 5 | 5+4+3 | -4.87*** | -4.87*** | 1.51 |  | 114 | 4 | 4 | -3.25*** | -5.06*** | 2.17* |
| *Rhododendron mucronulatum* | Ericaceae | 124 | 5 | 4+3 | -2.34*** | -3.71*** | 2.32* |  | 121 | 5 | 4 | -2.66*** | -4.28** | 2.35* |
| *Quercus mongolica* | Fagaceae | 127 | 5 | 4 | -3.67*** | -5.11*** | 0.36 |  | 136 | 5 | 4 | -4.64*** | -5.69** | -0.28 |
| *Juglans mandshurica* | Juglandaceae | 132 | 5 | 5+4+3 | -4.25*** | -4.25*** | 0.12 |  | 141 | 5 | 4 | -0.88 | -1.31 | 0.2 |
| *Lespedeza bicolor* | Leguminosae | 138 | 5 | 2+1+12 | -3.61** | 0.73 | -0.82 |  | 186 | 7 | 8+9 | -3.83** | -2.66 | -0.6 |
| *Albizia kalkora* | Leguminosae | 135 | 5 | 1 | -1.49* | 0.03 | 1.01 |  | 182 | 7 | 5+4 | -3.65* | -3.65* | 0.89 |
| *Gleditsia japonica* | Leguminosae | 130 | 5 | 5+4 | -0.96 | -0.79 | 0.58 |  | 163 | 6 | 6+5+4 | -6.08 | -2.41 | 1.21 |
| *Caragana arborescens* | Leguminosae | 128 | 5 | 4 | -2.76*** | -4.02*** | 0.03 |  | 138 | 5 | 5+4 | -3.11*** | -3.11*** | 0.75 |
| *Caraganarosea* | Leguminosae | NA | NA | NA | NA | NA | NA |  | 134 | 5 | 4 | -2.67** | -3.64** | 1.56 |
| *Morus alba* | Moraceae | 139 | 5 | 8 | -3.97** | -1.48 | -0.45 |  | 142 | 5 | 5+4 | -3.1** | -3.1** | 0.06 |
| *Syringa oblatavar.alba* | Oleaceae | 124 | 5 | 2+1+12 | -1.5** | -0.1 | 0.76 |  | 130 | 5 | 4 | -2.54*** | -3.59*** | 1.57* |
| *Forsythia mandschurica* | Oleaceae | 124 | 5 | 5+4+3 | -4.82*** | -4.82*** | 1.23 |  | 111 | 4 | 4 | -2.81*** | -4.33*** | 1.67* |
| *Ligustrum longipedicellatum* | Oleaceae | 123 | 5 | 3+2+1 | -2.84* | -0.59 | 1.09 |  | 169 | 6 | 6+5+4 | -3.21** | -2.08* | 0.12 |
| *Fraxinus mandschurica* | Oleaceae | 134 | 5 | 8 | -3.64* | 0.29 | -0.63 |  | 126 | 5 | 8 | -3.59* | -1.59 | 0.19 |
| *Syringa oblata* | Oleaceae | 125 | 5 | 5+4+3 | -4.16*** | -4.16*** | 0.91 |  | 131 | 5 | 4 | -3.05*** | -4.73*** | 1.05 |
| *Prinsepia sinensis* | Rosaceae | 106 | 4 | 4+3 | -3.21*** | -4.60*** | 1.31 |  | 109 | 4 | 4 | -2.71* | -4.05** | 2.17** |
| *Rosa xanthina* | Rosaceae | 123 | 5 | 4+3 | -2.84*** | -4.50*** | 1.16 |  | 149 | 5 | 5+4 | -3.72** | -3.72*** | 0.07 |
| *Pyrus ussuriensis* | Rosaceae | 125 | 5 | 5+4+3 | -4.42*** | -4.42*** | 0.76 |  | 125 | 5 | 4 | -3.4*** | -4.38*** | 1.63 |
| *Armeniaca sibirica* | Rosaceae | 132 | 5 | 4 | -2.66*** | -3.52*** | 0.76 |  | 124 | 5 | 4 | -2.98*** | -4.04*** | 1.48 |
| *Crataegus pinnatifida* | Rosaceae | 125 | 5 | 4+3+2 | -2.58*** | -3.69*** | 0.87 |  | 145 | 5 | 5+4 | -3.31*** | -3.31*** | 1.04 |
| *Spiraea chamaedryfolia* | Rosaceae | 110 | 4 | 4+3 | -2.56*** | -4.02*** | 2.29. |  | 130 | 5 | 4 | -2.43** | -4.35** | 1.88. |
| *Cerasustomentosa* | Rosaceae | 127 | 5 | 4 | -2.17*** | -3.23*** | 2.00* |  | 124 | 5 | 4 | -2.12** | -3.9** | 2.06* |
| *Amygdalus triloba* | Rosaceae | 129 | 5 | 5+4+3 | -4.2*** | -4.2*** | 0.68 |  | 125 | 5 | 4 | -3.23*** | -4.63*** | 1.31 |
| *Sorbaria sorbifolia* | Rosaceae | 110 | 4 | 4 | -3.02*** | -4.36*** | 0.16 |  | 191 | 7 | 10+9 | -5.29** | -3.41 | -2.11 |
| *Sorbuspohuashanensis* | Rosaceae | NA | NA | NA | NA | NA | NA |  | 140 | 5 | 5+4 | -3.78** | -3.78** | 1.43 |
| *Phellodendron amurense* | Rutaceae | 134 | 5 | 8 | -3.72*** | -1.3 | 0.04 |  | 158 | 6 | 5+4 | -2.3** | -2.30** | -0.27 |
| *Salix matsudana* | Salicaceae | 122 | 5 | 4+3 | -2.7*** | -3.89*** | 1.79. |  | 119 | 4 | 4+3 | -2.68*** | -4.56*** | 2* |
| *Xanthoceras sorbifolium* | Sapindaceae | 137 | 5 | 9+8 | -4.27*** | -0.25 | -0.63 |  | 141 | 5 | 5+4 | -4.26*** | -4.26*** | 0.37 |
| *Philadelphus schrenkii* | Saxifragaceae | 121 | 4 | 4 | -2.92*** | -3.90*** | 0.8 |  | 151 | 5 | 5+4 | -2.4** | -2.40** | -0.06 |
| *Ribes giraldii* | Saxifragaceae | 112 | 4 | 4+3 | -2.58*** | -3.84*** | 2.85* |  | 131 | 5 | 4 | -2.95** | -5.0* | 2.32. |
| *Ribes odoratum* | Saxifragaceae | 119 | 4 | 4+3 | -2.17*** | -3.18*** | 0.99 |  | 132 | 5 | 4 | -2.37*** | -3.67** | 1.57* |
| *Tiliamandshurica* | Tiliaceae | 126 | 5 | 9 | -7.65*** | -2.29 | -0.84 |  | 178 | 6 | 6+5 | -3.28** | -0.04 | -0.79 |
| *Tilia amurensis* | Tiliaceae | 129 | 5 | 1+12+11 | -2.75** | -0.97 | 0.82 |  | 178 | 6 | 6+5 | -2.26** | -1.26 | -0.77 |
| *Hemiptelea davidii* | Ulmaceae | 126 | 5 | 5+4+3 | -5.16*** | -5.15*** | 0.83 |  | 138 | 5 | 5+4+3 | -3.99*** | -4.49*** | 0.98 |
| *Ulmus davidiana* | Ulmaceae | 126 | 5 | 4 | -2.99*** | -4.01*** | 0.58 |  | 109 | 4 | 4 | -2.33* | -3.33* | 2.31. |
| *Ulmus pumila* | Ulmaceae | 125 | 5 | 4 | -2.98*** | -3.86*** | 0.73 |  | 107 | 4 | 4 | -3.53*** | -4.65*** | 2.68* |
| *Vitis amurensis* | Vitaceae | 130 | 5 | 3 | -1.44* | -1.48 | 1.74 |  | 145 | 5 | 12+11+10 | -6.42** | -4.08 | 0.97 |

Table S3. Results of ‘pgls (in the package ‘geigerin R) to compare whether the phenological responses to mean temperature of March, April, and May for leaf-out date and mean temperature of April and May for flowering date and winter precipitation was correlated with functional groups. ‘N’ is the number of species in each group. “-’ is empty value.

|  |  | Leaf-out phenology | | | | | | | |  | Flowering phenology | | | | | | | |
| --- | --- | --- | --- | --- | --- | --- | --- | --- | --- | --- | --- | --- | --- | --- | --- | --- | --- | --- |
|  |  | Temperature | | | |  | Winter precipitation | | |  | Temperature | | | |  | Winter precipitation | | |
| Group |  | N | t | R^2^ | P |  | t | R^2^ | P |  | N | t | R^2^ | P |  | t | R^2^ | P |
| Native status (native- vs. introduced-) |  | 38/7 | -1.43 | 0.05 | 0.16 |  | 1.62 | 0.06 | 0.11 |  | 40/7 | 2.10 | 0.09 | 0.04 |  | 0.97 | 0.02 | 0.34 |
| Pollination syndrome (animal- vs. wind-) |  | - | - | - | - |  | - | - | - |  | 37/10 | -0.61 | 0.01 | 0.55 |  | -0.17 | 0.001 | 0.87 |
| Fruit type (dry- vs. felshy-) |  | - | - | - | - |  | - | - | - |  | 26/21 | 0.62 | 0.01 | 0.54 |  | 1.00 | 0.02 | 0.32 |
| Earliness |  | 45 | 0.41 | 0.004 | 0.68 |  | -5.28 | 0.39 | <0.001 |  | 47 | -0.72 | 0.01 | 0.48 |  | -1.39 | 0.04 | 0.17 |
